# Genome-wide identification of the *Jatropha curcas* MYB family and functional analysis of the abiotic stress responsive gene *JcMYB2*

**DOI:** 10.1186/s12864-016-2576-7

**Published:** 2016-03-22

**Authors:** Xianjun Peng, Hui Liu, Dan Wang, Shihua Shen

**Affiliations:** Key Laboratory of Plant Resources, Institute of Botany, the Chinese Academy of Sciences, Beijing, 100093 China

**Keywords:** Abiotic and biotic stress, MYB transcription factor, Promoter, Tolerance

## Abstract

**Background:**

The MYB family is one of the most abundant transcription factor families in plants. MYB proteins are involved in plant development, abiotic stress tolerance, hormone signal transduction and disease resistance. Here we perform genome-wide identification of MYB family transcription factors in an energy plant *J. curcas*, including determining family composition, phylogenetic evolution and functional prediction analysis. In addition, we further elucidate the function of the *JcMYB2* gene.

**Methods:**

The phylogenetic trees were constructed by using the neighbor-joining method in MEGA 5.2. The biological functions of some JcMYBs were predicted according to orthology. The full length cDNA of *JcMYB2* was cloned by using the RACE method. GUS histochemical staining was used to test the activity of the *JcMYB2* promoter. Expression patterns of *JcMYB2* were detected by using qPCR Transcriptional activity JcMYB2 were confirmed through yeast one hybrid. Subcellular Localization of JcMYB2 Protein were demonstrated by transient expression in the tobacco leaf. The function of *JcMYB2* in salt and freezing tolerance were detected in transgenic plants.

**Results:**

A genome-wide analysis identified 128 MYB genes, including 123 R2R3-MYBs, 4 R1R2R3-MYBs and 1 4R-MYB. All of the R2R3-MYBs are further classified into 19 groups which indicated functional conservation among previously identified groups of R2R3-MYB proteins. Among of these newly identified MYBs, the JcMYB2 belongs to group G11 and its expression is induced obviously by cold, salt and MeJA (Methyl Jasmonate) and slightly by ABA (abscisic acid). JcMYB2 is localized to the nucleus and has transcriptional activity. *JcMYB2* overexpressing plants are more tolerant to salt and cold stress than wild type plants. Tissue specific expression profiles showed that the *JcMYB2* gene was expressed ubiquitously throughout the plant, with higher expression levels observed in the root.

**Conclusion:**

A comprehensive genome-wide analysis and phylogenetic relationship of R2R3-MYB subfamily in *J. curcas* present the global identification and functional prediction of JcR2R3-MYBs. Additionally, JcMYB2 regulates the stress response signaling networks by interacting with MeJA and ABA signaling pathway and functions in the root development of *J. curcas*.

**Electronic supplementary material:**

The online version of this article (doi:10.1186/s12864-016-2576-7) contains supplementary material, which is available to authorized users.

## Background

*J. curcas*, a deciduous tree species belonging to the Euphorbiaceae family, is a drought resistant oil species widely distributed in tropical and subtropical areas, especially in Central and South America, Africa, India and Southeast Asia [1]. Among the potential biofuel crops, *J. curcas* has been gaining importance as the most promising oilseed, as it does not compete with the edible oil supplies. However, the lack of adequate genetic variation and non-availability of improved varieties limits its prospects of being a successful energy crop [2]. In addition, *J. curcas* plants are endowed with a high tolerance to conditions of drought and heat, moderate tolerance to salinity and heavy metals, but very low tolerance to low temperatures [3] which is the bottleneck for cultivation and commercialization of *J. curcas*. Low temperatures have persistent and detrimental effects on *J. curcas* crop establishment via the depletion of photosynthesis brought by chilling and freezing injuries [4]. The damage of cold stress may cause a sharp decrease in chlorophyll contents and membrane unsaturated fatty acids of *J. curcas* and even survival of seedlings [1].

Gao et al. suggest that besides the ROS (Reactive oxygen species) scavenging system, flavonoids from the phenylpropanoid pathway can also protect membrane lipids during cold stress in *J. curcas* seedlings exposed to temperatures as low as 4 °C [5]. A global view of the *J. curcas* transcriptome in response to cold stress revealed that 4,185 transcripts are possibly associated with cold resistance [6]. In our previous study, we found that expression of the *JcERF* gene is rapidly induced upon salinity, drought, ethylene and mechanical wounding treatments. Overexpression of *JcERF* in transgenic *Arabidopsis* enhances the salt and freezing tolerance [7]. Additionally, expression of *JcDREB* is induced by cold, salt and drought stresses, but not by ABA. Transgenic *Arabidopsis* plants over-expressing *JcDREB* exhibit enhanced tolerance to salt and freezing stresses [8]. Although we have made some progress in understanding the molecular mechanisms of *J. curcas* cold stress responses, the roles of transcriptional factors, especially for the MYB family which play a conserved role in the regulation of stress responses, has not been well illustrated.

According to the number of adjacent MYB repeats (R), MYB proteins can be classified into four subfamilies: MYB proteins with one repeat are named 1R-MYB (or MYB-related proteins); with two, 2R-MYB (R2R3-MYB); with three, 3R-MYB (R1R2R3-MYB); and with four, 4R-MYB (R0R1R2R3-MYB) [9, 10]. In plants, R2R3-MYB is the largest subfamily. The phylogenetic comparison of this superfamily in *Arabidopsis* and rice indicated that the *Arabidopsis* MYB superfamily undergo a rapid expansion after its divergence from monocots but before its divergence from other dicots [11]. Currently, there is some controversy about the evolution and categorization of subgroups. For instance, in *Arabidopsis*, 126 R2R3-MYB have been listed into 25 subgroups by Dubos et al. [10], which is different from Kranz et al., who categorized these MYBs into 22 subgroups [12]. Nevertheless, there are similar functions between members of each subgroup and there is cross-interaction among of them [12]. Recently, the increasing availability of plant genome sequences has facilitated a better understanding of this large gene family. In addition to *Arabidopsis*, genome wide characterization of the MYB family, especially for R2R3-MYB, have been completed in rice [13], maize [14], barely [15], foxtail millet [16], soybean [17], orange [18] and apple [19]. Comparative expression profile analysis of R2R3-MYB genes in these species suggested that MYB proteins play conserved and various roles in development, growth and regulation of the metabolism of plants [10]. MYB proteins have important roles in phytohormone signal transduction and various stress response pathways [20, 21]. The expression of *AtMYB2* is up-regulated by ABA and the overexpression *AtMYB2* enhances the drought tolerance of transgenic plants [22]. Under these conditions, the *RD22* and *AtADH1* are also up-regulated, which suggests that AtMYB2 functions in ABA-inducible gene expression under drought stress in plants [22, 23]. By interacting with ICE1, AtMYB15 acts as a negative regulator of freezing tolerance by suppressing the expression of CBF [24]. GENEVESTIGATOR analysis show that 44.67 and 47.21 % MYB genes in *Arabidopsis* are up- and down-regulated in cold stress, respectively [13]. It is thus reasonable that MYB proteins may be key regulatory nodes of plant responses to cold stresses. Therefore, in this study, we performed genome-wide identification of the MYB family in *J. curcas*, and further determined the function of the cold and salt responsive *MYB2* gene in *J. curcas*.

## Results

### Identification of J. Curcas MYB genes

We used three approaches to identify the MYB protein encoding genes in *J. curcas* genome. To confirm the MYB protein models that were identified, all of the genes derived from the selected *JcMYB* candidate genes were examined using the online databases Pfam and SMART with default cutoff parameters. As a result, 123 typical non-redundant R2R3 MYB transcription factors, 4 R1R2R3 MYBs, and 1 4R-like MYB s were confirmed from the original data. This number is similar with that found in *Arabidopsis*. Additionally, the protein character of these 123 R2R3 MYB transcription factors were list in Additional file 1.

### Phylogenetic analysis and functional prediction of J. Curcas R2R3-MYB

**Fig. 1:**
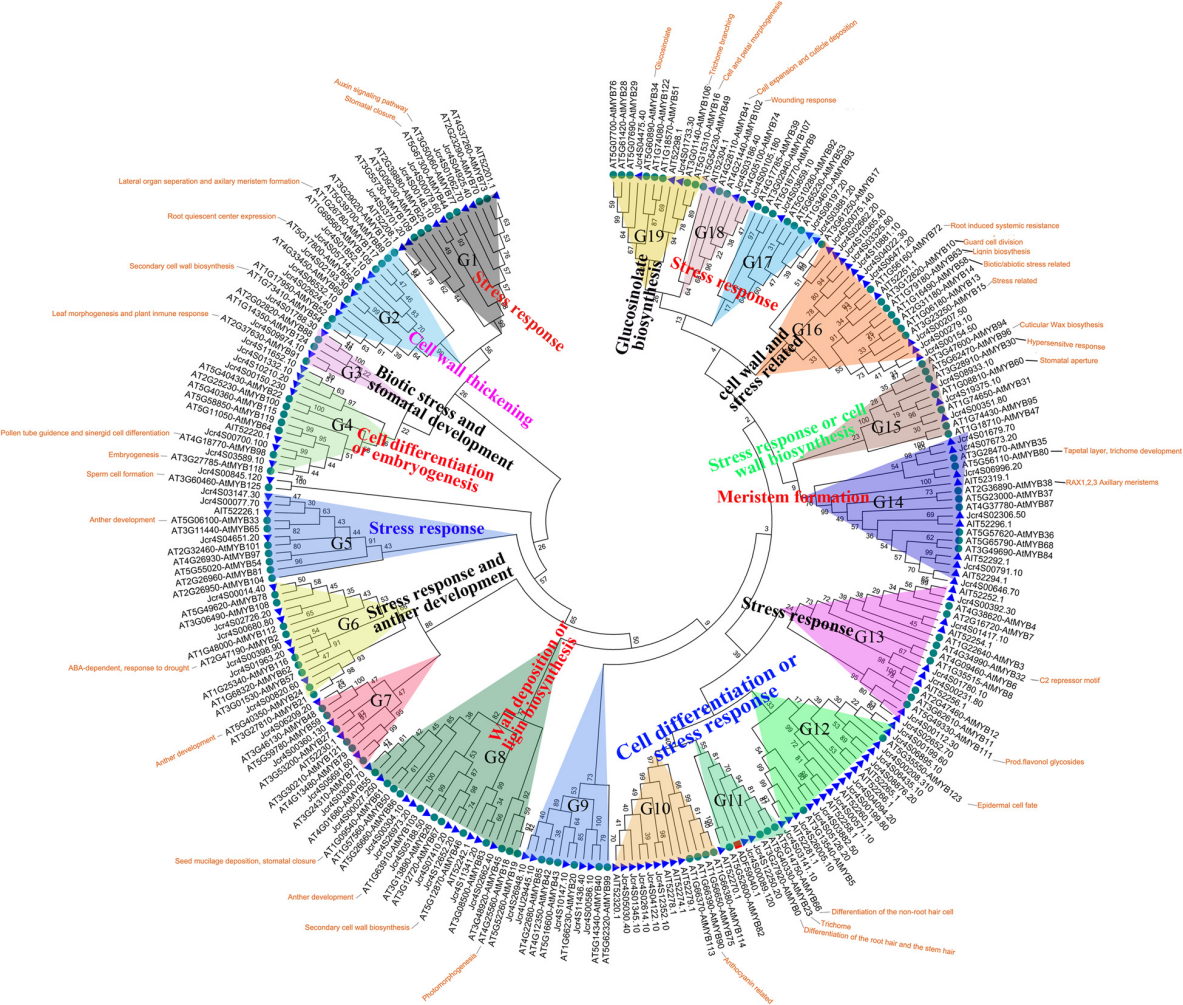
Evolutionary relationships of JcR2R3-MYB. The evolutionary history was inferred using the Neighbor-Joining method. The statistical method is Maximum Likelihood (ML). The bootstrap consensus tree inferred from 1000 replicates is taken to represent the evolutionary history of the MYBs analyzed. The evolutionary distances were computed using the p-distance method and are in the units of the number of amino acid differences per site. The analysis involved 249 amino acid sequences. Evolutionary analyses were conducted in MEGA5.2. Among of these, JcMYB2 is a member of group 11 and identified by the red square

To evaluate the evolutionary relationships within the R2R3-MYB gene family, we performed a combined phylogenetic analysis of *Arabidopsis* and *J. curcas* MYB proteins using the neighbor joining method. Based on conserved DNA binding domain similarity and topology of their encoded proteins, we subdivided the 123 typical members of the *J. curcas* R2R3-MYB gene subfamily into 19 groups (designated G1-G19) according to the alignment results (Fig. 1). The alignment result was provided in Additional file 2. Generally, the number of orthologs were similar between *Arabidopsis* and *J. curcas*. However, some homologs were clustered remarkably by species within the same clade or the number of homologs from two species was greatly asymmetrical in one group or clade. For example, two AtMYBs and 17 JcMYBs were included in group G12.

The functions of some *Arabidopsis* R2R3 MYB members have been well characterized experimentally, and phylogenetic analysis has identified several functional groups [18, 25, 26]. Most of JcR2R3-MYB proteins were clustered into the functional groups found in *Arabidopsis* (Fig. 1), including stress response, cell cycle, cell wall thickening, meristem formation, and secondary metabolism.

### Cloning and expression profile of the JcMYB2 gene

**Fig. 2:**
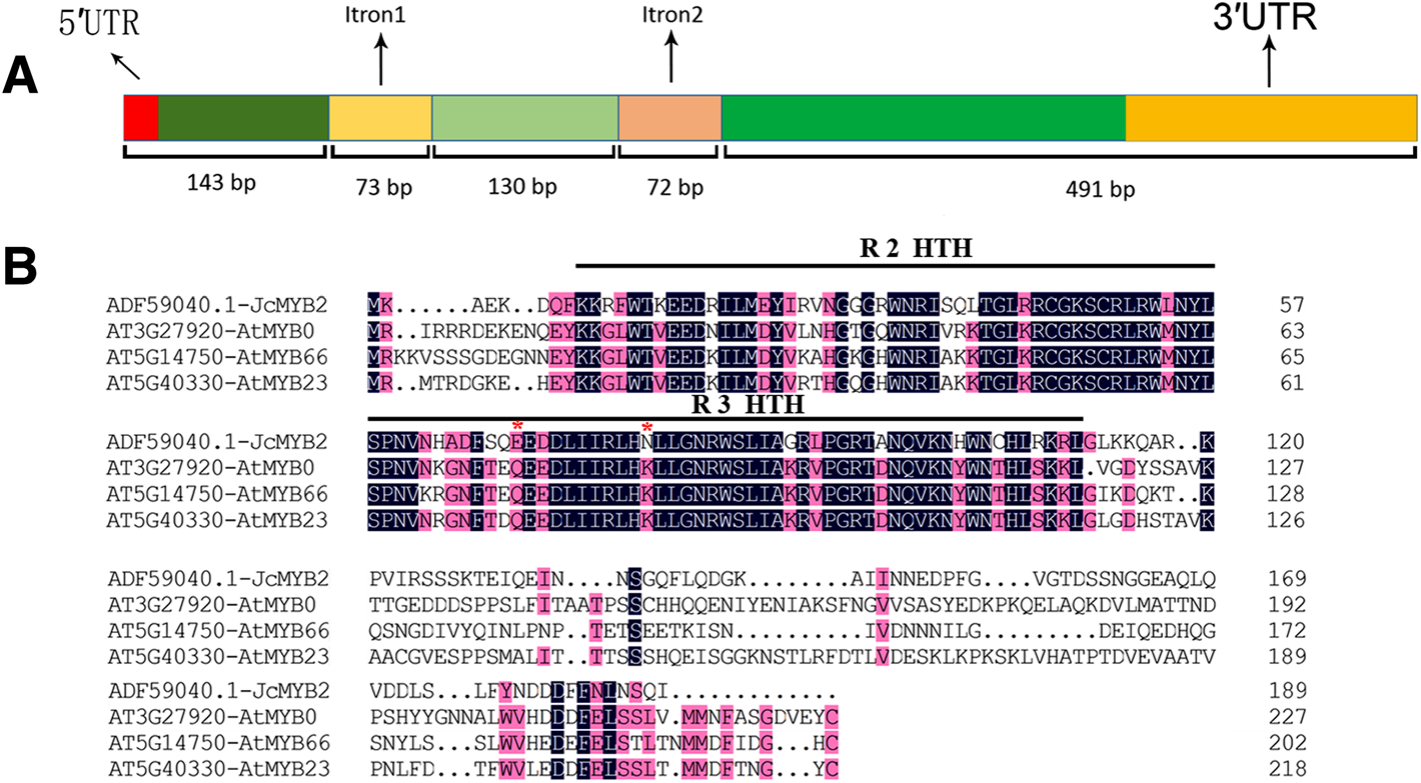
The gene structure and the dimensional structure of JcMYB2 protein. **a** The structure of *JcMYB2* gene. There were two introns and three exons in this gene. The length of exon 1 and 3 included the length of the 5′ and 3′ UTR, respectively. **b** The alignment of JcMYB2 and its ortholog proteins of Arabidopsis. The red asterisk show the variant amino acid residues. R2 and R3 HTH represent the MYB repeat domain

The full length of *JcMYB2* gene (GenBank accession: GU937787) is 908 bp and contains an ORF (open reading frame) of 570 bp that encodes a protein of 189 amino acids with a predicted molecular mass of 21. 9 kDa and a p*I* of 9.435. Analysis of the deduced amino acid sequence indicated that it is an R2R3-MYB protein with two imperfect repeat sequences in its MYB domain. Based on the phylogenetic tree of *J. curcas* and *Arabidopsis* MYB proteins, JcMYB2 clustered in group G11 with *Arabidopsis* AtMYB66, AtMYB23 and AtMYB0 (Fig. 1). There are two introns in the ORF of *JcMYB2*, with lengths of 73 and 72 bp, respectively. The gene structure of *JcMYB2* is shown in Fig. 2a.

The three-dimensional spatial structure of the JcMYB2 protein, predicted via CPHmodels-3.0, showed that JcMYB2 spatial structure has typical structure for R2R3-MYB proteins, namely two consecutive HTH (Helix-Turn-Helix) domains (Fig. 2b and Additional file 3); these constitute its DNA-binding domain and are predict to bind the major groove of DNA.

**Fig. 3:**
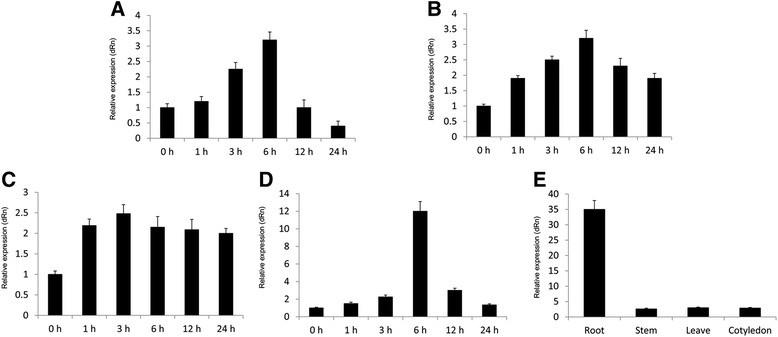
Expression patterns of *JcMYB2*. **a** Expression pattern of *JcMYB2* under 4 °C cultivate condition. **b** Expression pattern of *JcMYB2* under 200 mM NaCl treatment. **c** Expression pattern of *JcMYB2* under 100 μM ABA treatment. **d** Expression pattern of *JcMYB2* under 100 μM MeJA treatment. **e** The organ specific expression pattern of the *JcMYB2* gene. Transcript levels were determined by quantitative RT-PCR. Expression of *J. curcas* actin was used as an internal control. Error bars indicate standard deviation of three independent biological replications

To determine whether *JcMYB2* functions in stress responses in *J. curcas*, we assayed its expression following exposure of the plants to cold temperatures; high salt conditions; and both ABA and MeJA. Results showed that *JcMYB2* transcripts were induced after 3 h cold stress treatment and reached their maximum at 6 h (Fig. 3a). Its expression pattern under salt stress was similar with that of cold (Fig. 3b). For ABA treatment, expression of *JcMYB2* was initially induced after 1 h and accumulated continuously for 24 h (Fig. 3c). Additionally, under MeJA treatment *JcMYB2* transcript was gradually increased and also reached its maximum at 6 h under MeJA treatment, then decreased sharply and returned to original levels (Fig. 3d). Under normal conditions, the expression of *JcMYB2* in the root was much higher than that in the stem, leaf and cotyledon (Fig. 3e) which suggested that *JcMYB2* was differentially expressed within different tissues of the plant.

### Promoter isolation and activity analysis of JcMYB2

**Fig. 4:**
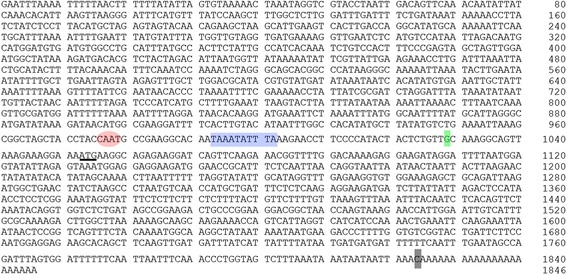
The sequence of *JcMYB2* gene. Start codon ATG is marked by underline, conserved region of TATA-box and CAAT region are indicated in blue box and red ellipse respectively. The transcriptional site and termination site are marked as green and gray box respectively

The promoter sequence of *JcMYB2* was cloned using Tail-PCR and was subsequently confirmed by PCR designed according to the *J. curcas* genome database (Fig. 4). The structure of the *JcMYB2* promoter region was analyzed by searching the plant *cis*-acting element database PLACE (Plant *Cis*-Acting regulatory DNA Elements) and Plant CARE (Plant *cis*-acting regulatory element database); the results are shown in Table 1. This promoter contains total putative 72 *cis*-elements classified in to 11 types, including those involved in ABA responses and stress tolerance, low temperature stress tolerance, JA-induced plant disease resistance, SA-induced pathogen resistance and meristem development.

**Table 1:** Predicted *cis*-acting elements of *JcMYB2* promoter

*Cis*-elements	Function of *cis*-elements	Number
ACGT	ABA responses and stress tolerance	11
CANNTG	Low temperature stress tolerance	10
TTGAC	JA induced plant disease resistance	11
TGTCA	SA induced pantheon resistance	4
AAAAG	Light response elements	10
ACCWWCC	MYB binding site	10
YACT	P starvation response	5
CATATG	Auxin responsive	3
GGTTAA	Meristem development	6
TGHAAARK	Endosperm development	4
GTGA	Late pollen development	2

**Fig. 5:**
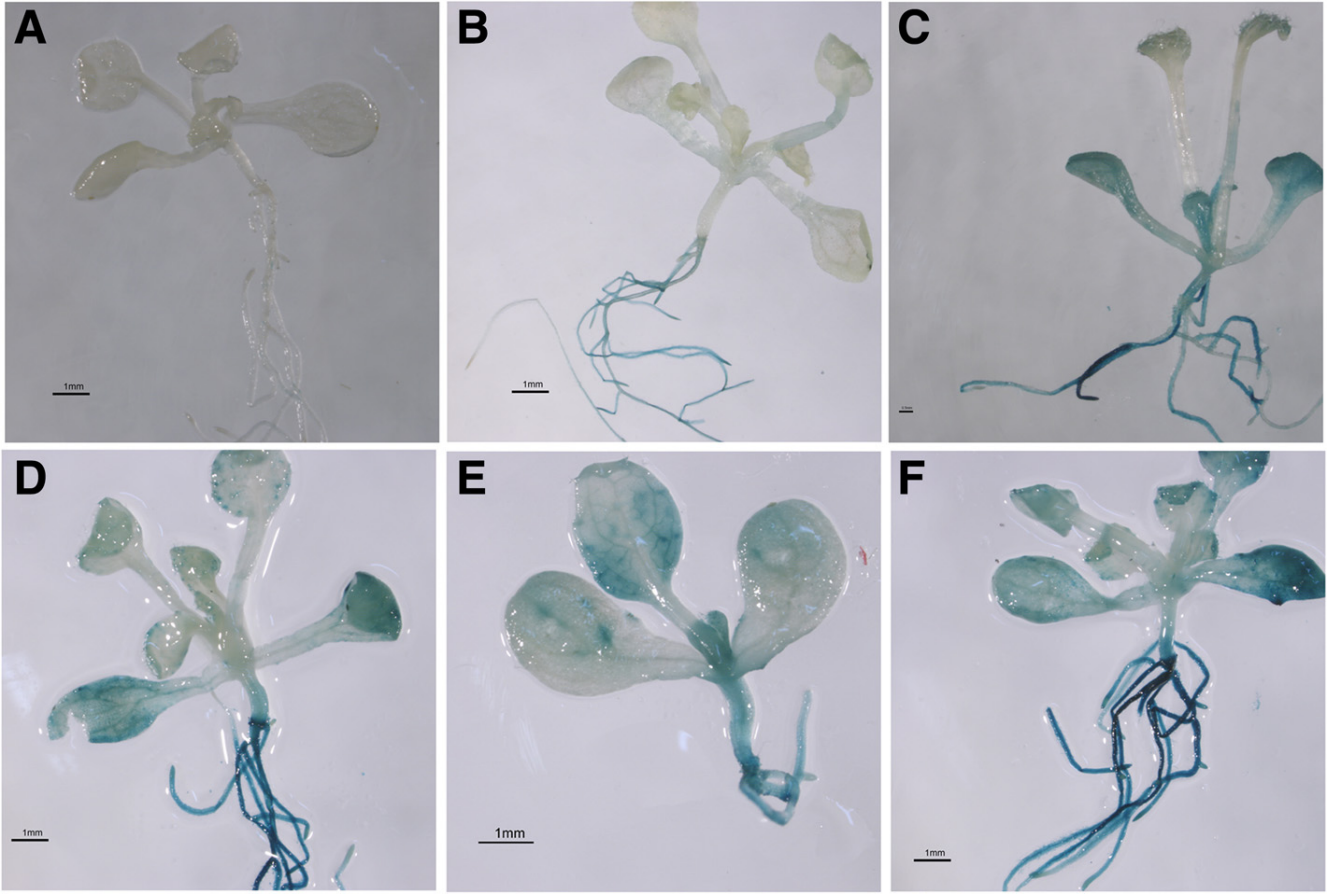
The promoter activity of *JcMYB2*. Detection of *JcMYB2* promoter activity by GUS staining. **a** wild type. **b** the transgenic plant under normal cultural condition. **c** seedlings under 4 °C cultivate condition for 6 h. **d** seedlings under salt treatment (treated with 200 mM NaCl for 6 h). **e** seedlings under ABA treatment (treated with 100 μM ABA for 3 h). **f** seedlings under MeJA treatment (treated with 100 μM MeJA for 6 h). B, C, D, E and F show GUS staining signal except for A. The GUS staining analysis of transgenic Arabidopsis harboring Pro-JcMYB2:: GUS revealed that the JcMYB2 promoter was active in transgenic seedlings. C, D, E and F display GUS staining signal which proved that its activity of JcMYB2 promoter will be increased under cold, salt, ABA and MeJA treatment

GUS staining of transgenic *Arabidopsis* harboring Pro-*JcMYB2*::GUS revealed that the *JcMYB2* promoter is very active in the root, but has only slightly activity in the leaf and stem (Fig. 5b). Cold, salt, ABA and MeJA could induce GUS expression in transgenic *Arabidopsis* (Fig. 5c-f).

### Transcriptional activity and subcellular localization of the JcMYB2 protein

**Fig. 6:**
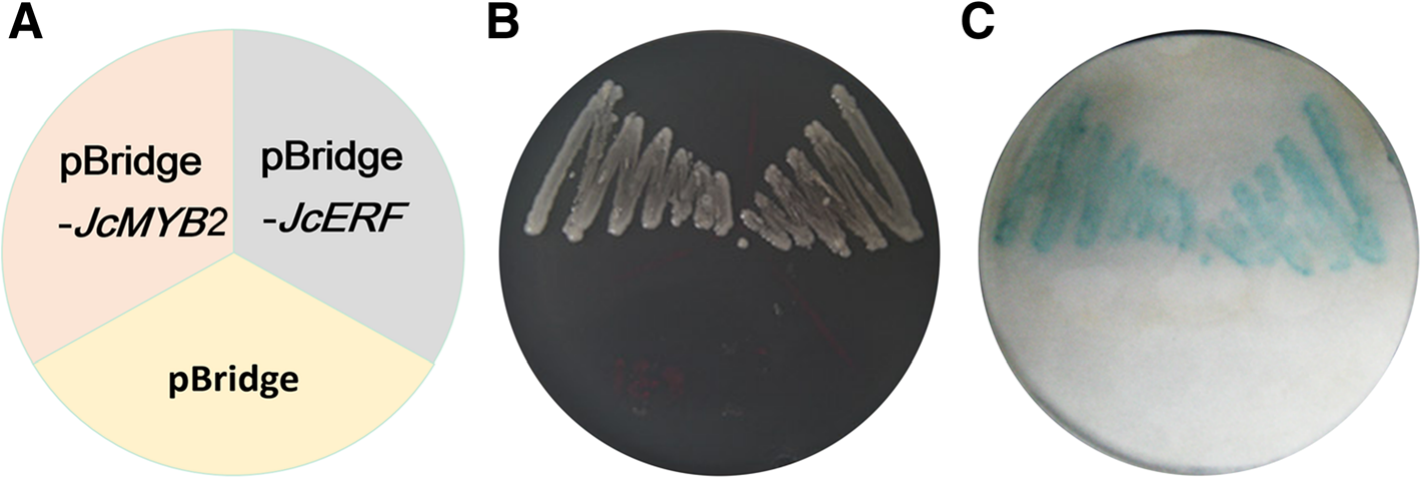
Transactivational assay of JcMYB2. The full-length ORF of JcMYB2 was fused with pBridge, and the transformed AH109 yeasts were selected from SD-Trp-His medium. JcERF was used as a positive control, and empty pBridge vector was used as a negative control. **a** The position of each transformed yeast cell. **b** The growth status of the transformed AH109 cells on the SD medium. **c** the reporter activity detected by β-galactosidase activity assay

The transcriptional activity of JcMYB2 was tested using a yeast one-hybrid assay. The yeast strain AH109 containing the vector pBridge (as a negative control) could not grow on SD medium without His and Trp (SD/-His-Trp). However, cells harboring the pBridge-JcMYB2 and pBridge-JcERF (as a positive control) could grow normally on the same medium and exhibited blue in β-galactosidase assay (Fig. 6a-c). These results indicate that JcMYB2 can function as a transcriptional activator.

**Fig. 7:**
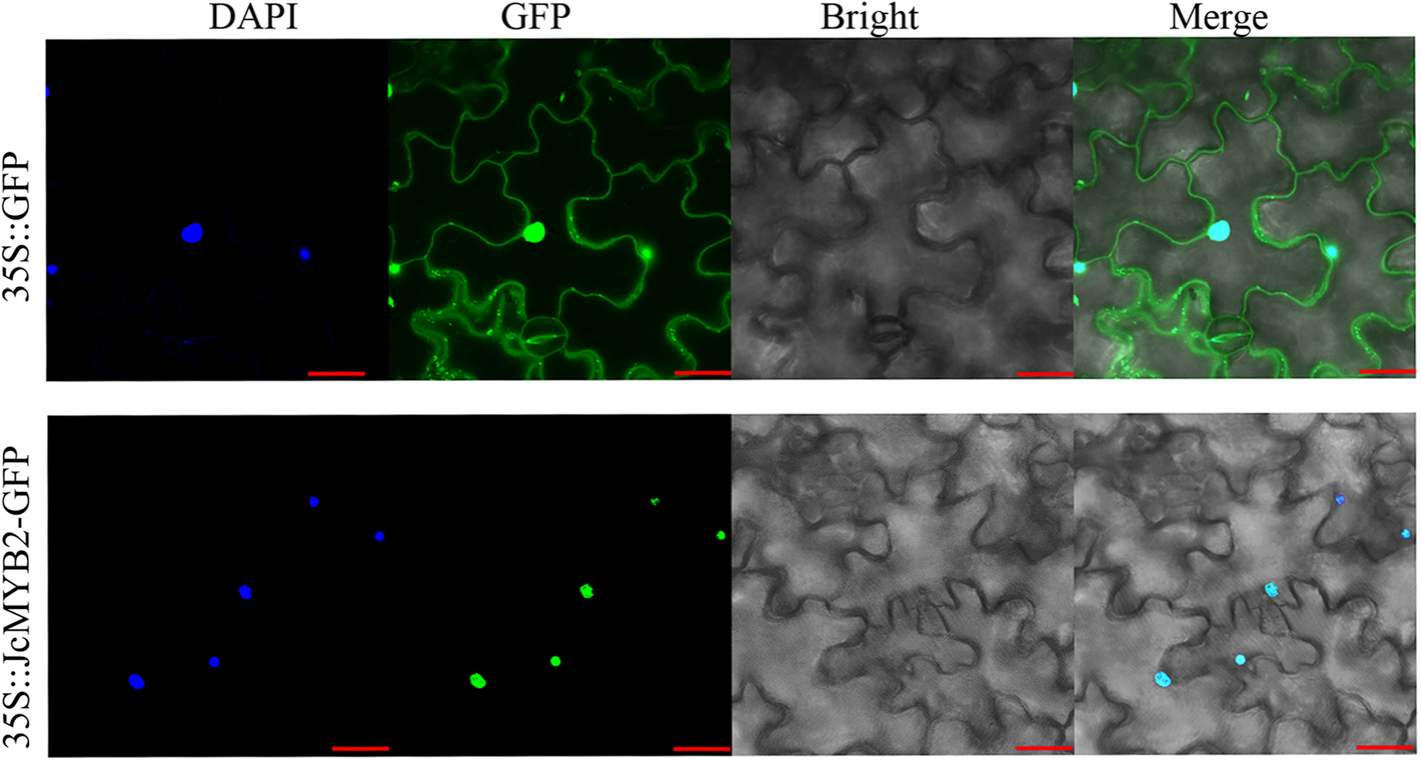
Subcellular localization of JcMYB2 protein. Transient expression of pCAMBIA1302- JcMYB2 fusion and pCAMBIA1302 construct in tobacco epidermal cells. Green fluorescence was observed using a confocal microscope at 48 h after *A. tumefaciens* infiltration. Micrographs showing cells expressing GFP (control, upper lane) or JcMYB2::GFP (bottom lane) fusion protein. From left to right, the pictures showed DAPI, fluorescent-field illumination, bright-field, and overlay of three illuminations. Bar = 30 μm

To determine the subcellular localization of JcMYB2, we visualized a JcMYB2-GFP fusion protein in tobacco epidermal cells using confocal microscopy. In these cells, GFP signal was observed exclusively in the nucleus, while GFP fluorescence in control, pCAMBIA1302-GFP was distributed in the whole cell (Fig. 7). Thus, the result suggested that JcMYB2 is a nuclear protein.

### Heterologous expression of JcMYB2 enhances salt and freezing tolerance of Arabidopsis

**Fig. 8:**
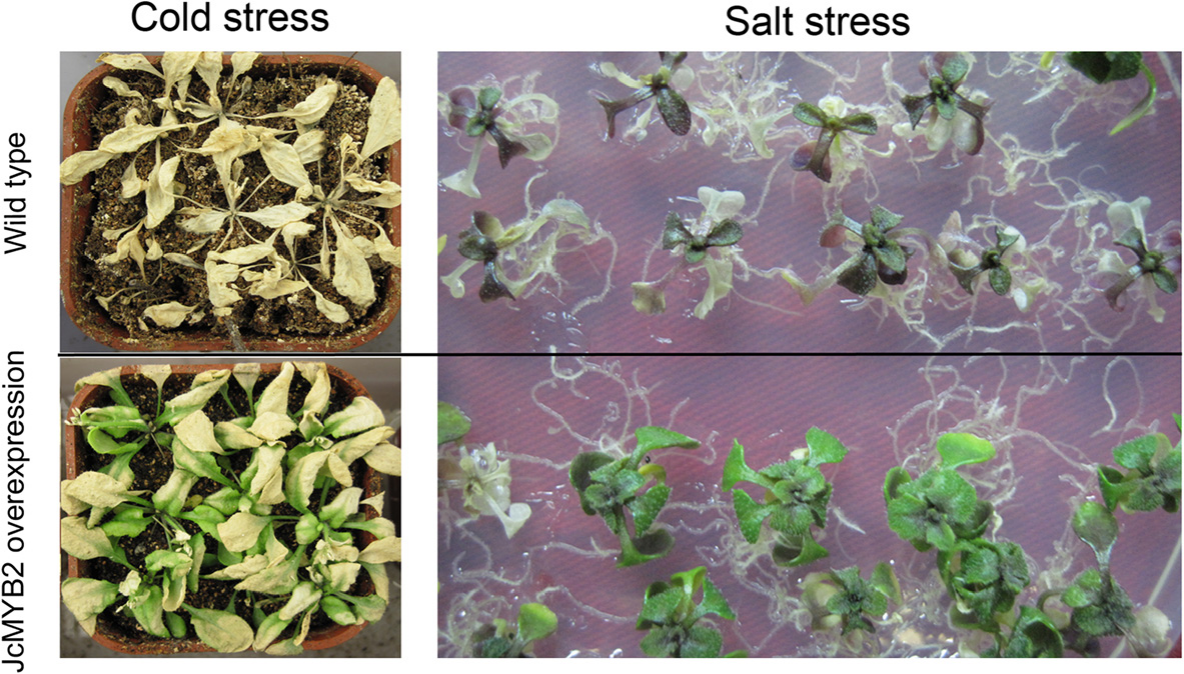
Cold and salt stress response of Arabidopsis transgenic plants overexpressing *JcMYB2*. Three-week-old plants, including wild type and transgenic plants, were exposed to −8 °C for 20 h, after which the temperature was returned to 23 °C for 7 days. The growth of Wild type and transgenic lines plants were grown on MS media for 10 days and then transferred onto the MS with 200 mM NaCl for salt stress. When the difference of phenotype between Wild type and transgenic lines were apparent, the photograph were taken

To investigate the biological function of *JcMYB2*, ten day old seedlings of transgenic and wild type *Arabidopsis* were transferred to MS medium supplemented with 200 mM NaCl after germination on MS medium. The survival percentage of *JcMYB2* transgenic *Arabidopsis* was more than 85.6 % under salt stress, which was significantly higher than that of wild type *Arabidopsis* (lower than 14.8 %) (Fig. 8). For the freezing tolerance test, three-week-old plants were exposed to −8 °C for 20 h, and then recovered in chambers at 23 °C for 7 d. The transgenic plants expressing *JcMYB2* were able to grow after being removed from sub-zero temperatures, while the wild type plants withered and died under these conditions (Fig. 8). Taken together, these results show that overexpression of *JcMYB2* in *Arabidopsis* confers tolerance to both high salt and low temperature stresses.

## Discussion

### Identification and functional prediction of the J. curcas MYB family

To identify the MYB transcription factor-encoding genes in *J. curcas*, we searched the entire *J. curcas* genome and two databases for genes that encode proteins containing the MYB DNA binding domain. As is well known, the MYB family is the most abundant transcription factor family in plants and R2R3-MYB is the largest subfamily. Correspondingly, there are 157 R2R3-MYB proteins in the maize [14], 100 in the sweet orange [18], 126 in *Arabidopsis*, 109 in rice [13], 252 in soybean [17] and 192 in *Populus* [27]. Additionally, there are 222 typical R2R3-MYB proteins, five R1R2R3-MYB proteins, and two 4R-like MYB proteins in the apple genome [19]. While the number of R2R3-MYB-encoding genes has expanded in different species, the number of R1R2R3-MYB and 4R-MYB genes has not changed. All vertebrate MYB proteins are in the 3R-MYB subfamily, which is also present in almost every plant studied [28], suggesting that the 3R-MYB encoding genes represent an ancient and evolutionarily conserved family. In both plants and vertebrates, 3R-MYB proteins regulate progress through cell cycle transitions [29].

These *J. curcas* R2R3-MYB proteins were divided into 19 groups, and the phylogenetic results was consistent with the recent reports [18, 26], which confirmed that our results placed the *J. curcas* proteins into the correct subfamilies. In general, the functions of members of the same clade seem highly but not absolutely conserved across different plants. The functions of some *Arabidopsis* R2R3-MYB proteins have been well characterized experimentally, and phylogenetic analysis has identified some functional groups [18, 25, 26]. Therefore, it is useful to identify the orthologs between plants based on their evolutionary relationships. Although the functions of most JcMYBs have yet to be characterized, all of the JcMYB proteins clustered into *Arabidopsis* functional groups (Fig. 1) which could provide useful information for predicting and studying the functions of JcMYBs within each clade. Many JcR2R3-MYBs were grouped into G13, G15, G17 and G18, whose members have been shown to be involved in stress response in *Arabidopsis* (Fig. 1), which thus facilitated identifying the JcMYBs that may play roles in the response to stress conditions.

### JcMYB2 is a typical R2R3-MYB transcription factor

We isolated one R2R3-*JcMYB2* gene from *J. curcas* by RACE PCR. The targeting experiment confirmed that JcMYB2 localizes to the nucleus (Fig. 7) and a yeast one hybrid assay suggested that JcMYB2 possesses transcriptional activation ability (Fig. 6). In addition, two introns of *JcMYB2* are located up- and down-stream of the sequence encoding the conserved R2R3 repeat domain (Fig. 2a), which was similar to its orthologous in *Arabidopsis*. Thus, we inferred that JcMYB2 might originate from the same ancestral gene as Jcr4S12250.20, AT3G27920 (AtMYB0) and AT5G14750 (AtMYB66), and some variation occurred in the DNA binding domain, though the length and position of the introns remain highly conserved.

Besides, JcMYB2 was clustered in the G11 with other MYB proteins from *Arabidopsis* (Fig. 2b), including AtMYB0, AtMYB23 and AtMYB66. The first helix of the second repeat domain is essential for the formation of the ternary complex [30, 31]. For AtMYB0 and AtMYB66, some variation of these residues, especially the mutation of the K residues will prevent their interaction with other proteins. According to the alignment of the full length protein of these four MYBs, in addition to the highly conserved amino acid residues, we observed that K is replaced by N residue in JcMYB2 (Fig. 2b). Additionally, there is another conserved residue Q is replaced by E in JcMYB2. Therefore, compared with AtMYB0 and AtMYB66, we hypothesized that JcMYB2 might play a different role in *J. curcas*.

### JcMYB2 is involved in growth regulation, development and tress tolerance

MYB proteins have been reported to be involved in response to cold stress in rice. For instance, overexpression of OsMYB4 significantly enhanced tolerance to chilling and freezing stress in transgenic *Arabidopsis* [32]. Ma et al. reported that OsMYB3R-2 participates in the cold signaling pathway by targeting the cell cycle and a putative DREB/CBF transcription factor [33]. Moreover, a recent study revealed that an R2R3-MYB gene, OsMYB2, is induced by salt, cold, and dehydration stress. *OsMYB2* overexpressing plants are more tolerant to salt, cold and more sensitive to abscisic acid than wild type plants [34]. Expression pattern analysis of the 156 MYB genes identified in soybeans suggests that expression of 43 of these genes change upon treatment with cold, salt and/or drought stress [35]. GmMYBJ1 displays similarities to the typical R2R3MYB proteins reported in other plants and overexpressing *GmMYBJ1* causes enhanced tolerance to drought and cold stresses, which indicates that GmMYBJ1 has the potential to be utilized in transgenic breeding lines to improve abiotic stress tolerance [36]. *PtsrMYB* isolated from the trifoliate orange shares the highest degree of identity with AtMYB109 and is up-regulated by abiotic stresses such as dehydration, salt, cold and ABA treatment [37].

The expression of *JcMYB2* was significantly enhanced under salt and cold stress, as well as induced by ABA and MeJA treatment. Therefore, we inferred that JcMYB2 is involved in ABA-dependent cold and salt tolerance and JA-mediated disease resistance signaling pathways. There is significant cross-talk among abiotic stress response mediated by ABA and the biotic stress response mediated by JA. *Arabidopsis* mutants with ABA insensitivity have the strong resistance against pathogens. On the other hand, excess exogenous ABA results in wild type plant susceptibility to the pathogens [38]. However, the infection will leads to an increase in the endogenous ABA [39]. Recent studies have reported that many kinds of transcription factors are induced expression by ABA and JA [40–45]. For instance, there is a report that the expression of *AtMYB2* and *AtMYC2* increases under ABA treatment, and their over-expression enhances the drought and salt tolerance [22]. Further studies have revealed that these genes also play important roles in the JA-mediated pathogen resistance signaling pathway, and *AtMYC2* especially has been considered the essential junction between biotic and abiotic stress [46, 47].

In plants, gene expression is determined by the promoter. An analysis of the *JcMYB2* promoter revealed several stress response elements, including those involved in low temperature stress tolerance, ABA responses and stress tolerance, JA-induced plant disease resistance and SA induced pathogen resistance. The expression pattern of *JcMYB2* and GUS staining analysis validated the potential function of these elements. It is a remarkable that 10 MYB binding sites present in the promoter *JcMYB2*, which implies that *JcMYB2* might be regulated by itself or other MYB proteins. Additionally, the transgenic expression of *JcMYB2* in *Arabidopsis* enhanced salt and cold tolerance in our study. Thus we inferred that JcMYB2 functions in the ABA-dependent and MeJA-mediated abiotic and biotic stress responses.

In addition, many growth and development related elements were also found in the *JcMYB2* promoter, such as light response elements, meristem development, endosperm development and late pollen development. In this study, the *JcMYB2* showed clear differential expression with high expression in the root and low expression in the stem, leaf and cotyledon. Interestingly, the *AtMYB66*, the *Arabidopsis* ortholog of *JcMYB2*, is highly expressed in the root and hypocotyl, and only lowly expressed in other organs of *Arabidopsis* seedlings. AtMYB66 regulates differentiation of the non-root hair cell in the root and the non-stomata cell in the hypocotyl [48, 49], AtMYB0, another ortholog of *JcMYB2*, also controls the differentiation of the root hair and the stem hair [50]. There is some similarity in the expression of *JcMYB2* and that of *AtMYB*0 and *AtMYB66*, which implies that JcMYB2 might have a similar function in the control of root cell differentiation, which need further investigation. Taken together, these results suggest that JcMYB2 is not only involved in cold and salt stress response, but also functions in MeJA-mediated biotic stress, and regulation of root development and growth. To some extent, these results also showed the feasibility of predicting protein functions through phylogenetic analysis.

## Conclusion

In conclusion, this study presents a genome-wide identification of the MYB gene family in *J. curcas*. A total of 123 R2R3-MYB proteins were phylogenetically classified into 19 distinct groups and putative function of R2R3-MYB proteins was assigned based on phylogenetic results. Additionally, *JcMYB2*, located in the nucleus and activated the downstream gene expression, could enhance the salt and cold resistance of the transgenic plants. The results presented here will be helpful for future studies of the biological functions of JcMYB proteins.

## Methods

### Identification and sequence analysis of MYB proteins in J. curcas

Three approaches were used to identify the members of the JcMYB gene family in *J. curcas*. First, a HMM profile of the MYB DNA-binding domain (PF00249) from the Pfam database (http://pfam.sanger.ac.uk/) was used for the identification of MYB genes in *J. curcas* genome (http://www.kazusa.or.jp/jatropha/). The default parameters were employed; the number of search results was set as 200. Second, we used MYB and *J. curcas* as keywords to search the NCBI (http://www.ncbi.nlm.nih.gov/) and all of the retrieved sequences were download. Third, the protein sequence and their DNA binding domain of MYB proteins were download from the Plant Transcription Factor Database (http://planttfdb.cbi.pku.edu.cn/). All of the protein sequences deriving from the selected MYB candidate genes were examined with the domain analysis programs of Pfam and SMART (http://smart.embl-heidelberg.de/) with default cutoff parameters. Finally, all of the protein sequences were compared with known MYB sequences by alignment to verify that the candidate sequences from *J. curcas* encoded MYB-containing proteins.

### Phylogenetic analysis and function prediction of the JcR2R3-MYBs

The *Arabidopsis* R2R3-MYB proteins and their conserved DNA binding domains were downloaded from the Plant Transcription Factor Database. The conserved DNA binding domain of R2R3-MYBs from *J. curcas* and *Arabidopsis* were aligned by MUSCLE in MEGA 5.2 and adjusted manually. The default parameters for gap open and gap extend is −2.9 and 0, respectively. Then, the phylogenetic trees were constructed based on the multiple sequence alignment by using the neighbor-joining method in MEGA 5.2 with 1000 bootstrap replicates. The images of the phylogenetic trees were drawn in MEGA5.2. Additionally, the biological functions of some JcMYBs were predicted based on the aforementioned phylogenetic tree according to orthology. In addition, the three-dimensional spatial structure of the JcMYB2 protein was predicted via CPHmodels-3.0.

### Plant materials and treatments

The seeds of *J. curcas* were grown in pots with 1:1(v/v) vermiculite vs peat medium and incubated at 28 °C with a 16 h light/8 h dark photoperiod for three weeks. For cold treatment, the seedlings were transferred into a 4 °C growth chamber. For salt and ABA treatment, the seedlings were washed carefully and transferred into the solutions of 200 mM NaCl and 100 μM ABA, respectively. For MeJA treatment, 100 μM solution was sprayed onto the surface of the seedlings. Both the control and stress treated seedlings were harvested at various periods (1, 3, 6, 12 and 24 h), flash frozen in liquid nitrogen and stored at −80 °C for further analysis. The root, stem, leaf and cotyledon were collected for tissue expression analysis.

### Isolation of the JcMYB2 gene and promoter

Total RNA was extracted from *J. curcas* samples as described by Tang [8] and digested by RNase-free DNase I (TaKaRa, Japan) to remove the genomic DNA. The genomic DNA of *J. curcas* was extracted using a DNAsecure PlantKit (Tiangen Biotech, Beijing). The concentrations of DNA and total RNA were determined using a Nano Drop cDNA for amplification of the 5′ and 3′ ends of *JcMYB2* was prepared using a SMARTTM RACE cDNA Amplification Kit (Clontech, Japan). The RACE primers of *JcMYB2* were designed based on the results of Sanger sequencing. The 5′ and 3′ end of *JcMYB2* were cloned following the manufacturer’s instructions. The reaction conditions were as follows: 94 °C for 4 min; 36 cycles of 94 °C for 30 s, 68 °C for 30 s, 72 °C for 50 s; followed by 72 °C for 10 min. The RACE results was provided in Additional file 4. Full-length *JcMYB2* cDNA was amplified using cDNA synthesized for the 3′ RACE as a template. The genomic sequences of *JcMYB2* were cloned using the same primers. The promoter of *JcMYB2* was cloned by using the TAIL PCR (thermal asymmetric interlaced PCR) with a Genome Walking Kit (TaKaRa, Japan). All the PCR products were cloned into the pMD19-Tvector (TaKaRa, Japan) and sequenced by Sangon Biotech (Shanghai).

To determine the activity of the *JcMYB2* promoter, the CaMV 35S promoter of pCAMBIA1301 was replaced by the promoter of *JcMYB2* to promote the expression of GUS. The recombinant plasmid was transformed into *Agrobacterium tumefaciens* EHA105, which was used to infect *Arabidopsis*. GUS histochemical staining was used to test the activity of the *JcMYB2* promoter. All of the primers were list in Additional file 5.

### Expression patterns of JcMYB2

Real-time quantitative PCR (qPCR) was completed on a qPCR machine (MX3000p, Strategene, CA, USA) using the SYBR PrimeScript RT-PCR Kit (TaKaRa), and the reaction condition was following: program of 40 cycles (95 °C for 5 s and 60 °C for 20 s). The 2^−ΔΔCT^ method was used to analyze the data and expression of *J. curcas* actin was monitored as an internal control. Information about the other primers used in qPCR can be found in Additional file 5.

### Transcriptional activity investigation

The entire coding region of *JcMYB2* was inserted into the *BamH*I and *Sal*I sites of pBridge to yield a fusion protein in frame with the GAL4 DNA binding domain. The primers used for PCR are listed in Additional file 6. The recombinant plasmid was sequenced to ensure that it could express proteins accurately. The recombinant plasmid was transformed into yeast AH109 with the reporter genes *His3* and *LacZ* according to the manufacturer’s instructions (Clontech, Palo Alto CA, USA). pBridge-JcERF [8] was used as a positive control. The empty pBridge vector served as a negative control. Transformed yeasts were cultured on SD medium without His and Trp. Subsequently, in order to test the expression of the *LacZ* gene, a colony lift filter assay was performed using o-nitrophenyl-β-D-galactopyranoside (ONPG) as a substrate to determine whether the JcMYB2 protein possesses transcriptional activity.

### Subcellular localization of JcMYB2 protein

The subcellular localization of JcMYB2 protein was determined as described by Peng [44]. The entire coding region of the target gene without a stop codon was amplified by PCR and inserted into the *Nco*I and *Spe*I sites of the vector pCAMBIA1302, which fuse GFP, in frame, to the 3′ end of *JcMYB2*. pCAMBIA1302 -JcMYB2-*GFP* construct was introduced into *Agrobacterium tumefaciens* strain EHA105. The tobacco leaf was infiltrated by the *Agrobacterium* to express the GFP fusion protein. Following incubation in growth chambers for 48 h, and then the injected leaf was stained by 100 ng/ml DAPI (4′–6-diamidino-2-phenylindole). After that, DAPI and GFP fluorescence in tobacco leaves was imaged by confocal scanning microscope (Leica, Göttingen, Germany).

### Analysis of salt and freezing tolerance in transgenic plants

The full-length *JcMYB2* cDNA was ligated into p3301-121 (reconstructed by our Lab) under the control of the CaMV 35S promoter. Empty vector was used as a control. The recombinant plasmid was transformed into *Agrobacterium tumefaciens* EHA105. *Arabidopsis* were transformed by the floral dip method. The T_0_ seeds were harvested, dried at 25 °C, and plated on half-strength sterile MS medium with 10 mg/l PPT selection pressure; subsequently surviving T_1_ seedlings were transferred to soil to set T_2_ seeds. T2 seeds and seedlings were used for the following analysis of salt and freezing tolerance.

Transgenic plants were grown in 8 cm pots filled with a mixture of peat/vermiculite (1:1, v/v) under light at 23 °C for 4 weeks. Three-week-old plants were exposed to freezing stress. Freezing stress was conducted by exposure of plants to a temperature of −8 °C for 20 h, after which the temperature was returned to 23 °C for 7 days. Transgenic plants were grown on MS media for 10 days and then transferred onto the MS with 200 mM NaCl for culture.

## Additional files

Additional file 1: The character of these 123 R2R3 MYB proteins. (XLSX 38 kb) Additional file 2: The alignment file of MYB binding domain of Arabidopsis and *Jatropha curcas*. Alignment analyses were conducted in MEGA5.2. (FAS 95 kb) Additional file 3: The predicted three dimensional structure of JcMYB2. Arrow: β-strand; Helix: α-helix; Single line: Turn. (TIF 800 kb) Additional file 4: The sequence of the RACE PCR. (XLSX 9 kb) Additional file 5: The composition of MYB proteins in plants. (XLSX 10 kb) Additional file 6: Primers used in this study. (XLSX 9 kb)
