# Supplementary figures and images for: Genome-wide identification of the Jatropha curcas MYB family and functional analysis of the abiotic stress responsive gene JcMYB2
Source: BMC Genomics. 2016 Mar 22;17:251. doi: 10.1186/s12864-016-2576-7 (PMC4804483; doi:10.1186/s12864-016-2576-7)

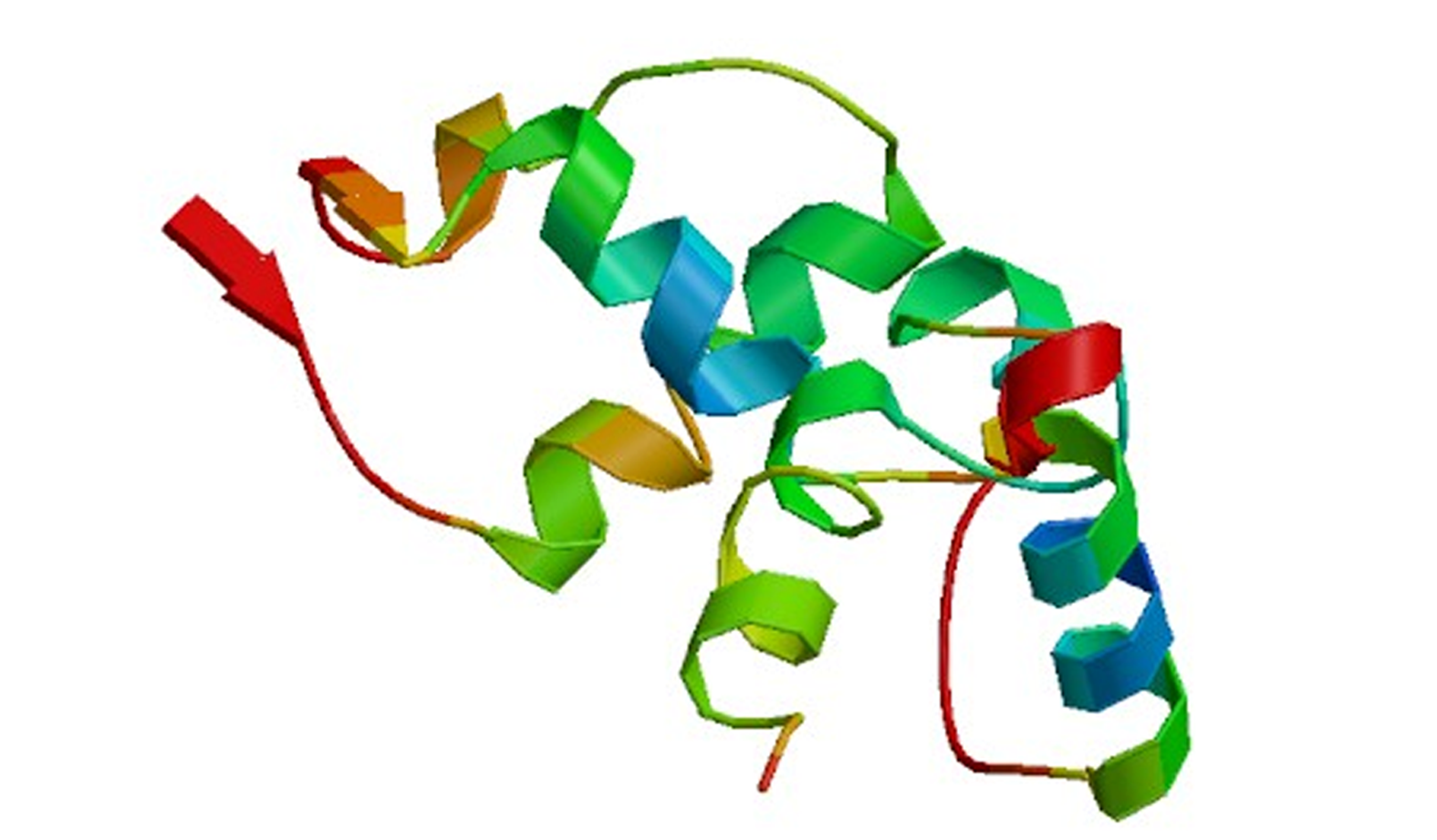

Supplement: Additional file 3: — The predicted three dimensional structure of JcMYB2. Arrow: β-strand; Helix: α-helix; Single line: Turn. (TIF 800 kb) [file 12864_2016_2576_MOESM3_ESM.tif]
